# Alpha2-Containing Glycine Receptors Promote Neonatal Spontaneous Activity of Striatal Medium Spiny Neurons and Support Maturation of Glutamatergic Inputs

**DOI:** 10.3389/fnmol.2018.00380

**Published:** 2018-10-15

**Authors:** Joris Comhair, Jens Devoght, Giovanni Morelli, Robert J. Harvey, Victor Briz, Sarah C. Borrie, Claudia Bagni, Jean-Michel Rigo, Serge N. Schiffmann, David Gall, Bert Brône, Svetlana M. Molchanova

**Affiliations:** ^1^Laboratory of Neurophysiology, ULB-Neuroscience Institute, Université Libre de Bruxelles, Brussels, Belgium; ^2^BIOMED Research Institute, University of Hasselt, Hasselt, Belgium; ^3^School of Health and Sport Sciences, University of the Sunshine Coast, Sippy Downs, QLD, Australia; ^4^Sunshine Coast Health Institute, Birtinya, QLD, Australia; ^5^Center for Human Genetics and Leuven Research Institute for Neuroscience and Disease, KU Leuven, Leuven, Belgium; ^6^VIB Center for the Biology of Disease, Leuven, Belgium

**Keywords:** autism spectrum disorders, dorsal striatum, medium spiny neurons, glycine receptors, spontaneous activity, synaptic development

## Abstract

Glycine receptors (GlyRs) containing the α2 subunit are highly expressed in the developing brain, where they regulate neuronal migration and maturation, promote spontaneous network activity and subsequent development of synaptic connections. Mutations in *GLRA2* are associated with autism spectrum disorder, but the underlying pathophysiology is not described yet. Here, using *Glra2*-knockout mice, we found a GlyR-dependent effect on neonatal spontaneous activity of dorsal striatum medium spiny neurons (MSNs) and maturation of the incoming glutamatergic innervation. Our data demonstrate that functional GlyRs are highly expressed in MSNs of one-week-old mice, but they do not generate endogenous chloride-mediated tonic or phasic current. Despite of that, knocking out the *Glra2* severely affects the shape of action potentials and impairs spontaneous activity and the frequency of miniature AMPA receptor-mediated currents in MSNs. This reduction in spontaneous activity and glutamatergic signaling can attribute to the observed changes in neonatal behavioral phenotypes as seen in ultrasonic vocalizations and righting reflex. In adult *Glra2*-knockout animals, the glutamatergic synapses in MSNs remain functionally underdeveloped. The number of glutamatergic synapses and release probability at presynaptic site remain unaffected, but the amount of postsynaptic AMPA receptors is decreased. This deficit is a consequence of impaired development of the neuronal circuitry since acute inhibition of GlyRs by strychnine in adult MSNs does not affect the properties of glutamatergic synapses. Altogether, these results demonstrate that GlyR-mediated signaling supports neonatal spontaneous MSN activity and, in consequence, promotes the functional maturation of glutamatergic synapses on MSNs. The described mechanism might shed light on the pathophysiological mechanisms in *GLRA2*-linked autism spectrum disorder cases.

## Introduction

Human subjects suffering from psychiatric disorders, including autism spectrum disorder (ASD) show defective connectivity in different brain regions and in many cases these disturbances in the neuronal network have a developmental origin (Yamasaki et al., 2017). Establishing the connectivity of neuronal networks is a process that is temporally and spatially regulated. For example, the formation and maturation of synaptic connections relies on recurring formation of neuronal protrusions (neurites and synapses). Furthermore, the activity of the neuron and local network in response to cell intrinsic programs and/or sensory experience are key regulators of synaptogenesis (Jamann et al., 2018). These findings have sparked an increased effort to characterize mechanisms that influence neuronal activity in the developing brain. Formation and refinement of glutamatergic synaptic connections occurs under the guidance of spontaneous electrical activity of new-born neurons (Cherubini et al., 2011; Huupponen et al., 2013). Activity-dependent maturation of synaptic connectivity has been described for many brain structures, including striatum (Dehorter et al., 2011; Kozorovitskiy et al., 2012). This activity propagates throughout the newly formed synaptic contacts, controlling post-translational modifications, promoting localized protein synthesis within dendrites and influencing gene transcription (Ebert and Greenberg, 2013). Mutations in genes that control these neuronal activity-dependent processes affect the maturation of synaptic connections (Lein et al., 2017).

Many neurotransmitter receptors, including glycine receptors (GlyRs), are known to be important regulators of neonatal spontaneous activity (Piton et al., 2011; Pilorge et al., 2016). GlyRs are ligand-gated chloride channels that are widely expressed throughout the brain and spinal cord. The diversity of the GlyRs results from a differential expression of four α-subunits (α1-α4) and one β-subunit (Malosio et al., 1991) and the composition of the GlyRs differs between brain regions and developmental age (Avila et al., 2013a). GlyRs containing the α2 subunit are highly expressed in the developing brain, where they act as depolarizing neurotransmitter receptors (Kilb et al., 2002). They regulate migration and maturation of cortical neurons and promote neonatal spontaneous neuronal network activity, which is needed for the development of synaptic connections (Avila et al., 2013b, 2014). Several mutations in *GLRA2* have been found in males with ASD, pointing at possible involvement of glycinergic transmission in development of cognitive abilities in these individuals (Pilorge et al., 2016). In a mouse model, deletion of *Glra2* causes alterations in glutamatergic circuitry and synaptic plasticity in the cerebral cortex (Morelli et al., 2016).

We previously reported that GlyRs are expressed in adult striatal projection neurons (i.e., medium spiny neurons, MSNs) (Molchanova et al., 2018) that contain α2 as the main agonist-binding subunit and are tonically active at resting state. The main function of these GlyRs is to stabilize the resting membrane potential (RMP) and set the offset of action potential firing. In the present study, we aimed to evaluate the expression profile of GlyRs in the developing striatum and studied their effect on intrinsic cellular MSN parameters relating to the maturation and formation of glutamatergic synaptic connections.

## Materials and Methods

### Animals

All animal experiments were conducted according to the ethical guidelines (in accordance with EU directive 2010/63/EU) provided by the University of Hasselt, the Université Libre de Bruxelles and the KU Leuven. The reduction and refinement of the animals experiments was achieved by an *ad hoc* power calculation (N) and designing paired experiments if possible. C57Bl/6 (both genders) and GlyR α2 subunit knockout mice (WTs and GlyRα2KOs, males) of specific ages were used in the study. GlyRα2KOs were initially generated in the laboratory of Prof. Harvey and Prof. T. N. Dear by deletion of the exon 7 of *Glra2*. GlyRα2KOs were fully back-crossed to C57Bl/6 background and genotyped as described previously (Avila et al., 2013a; D’Angelo et al., 1995).

### Immunohistochemistry, Imaging, and Analysis

Neuronal identification and morphological analysis were performed by biocytin injection into cells after whole-cell patch-clamp recordings. Slices were fixed overnight in 4% PFA, after which they were washed with 0.01 M phosphate-buffered saline (PBS in mM: 137 NaCl, 2.7 KCl, 10 NaH_2_PO_4_, 1.8 KH_2_PO_4_) and permeabilized with 0.01 M PBS 0.1% Triton-X 100 (Sigma-Aldrich) for 1.5 h. Streptavidin Conjugate (1:500; A488; Life Technologies) was added to the permeabilization solution and incubated for 4 h. Dendritic trees were imaged with a Plan-Apochromat 20×/0.75 objective, resolution of 2.3 pixels/μm and Z-stack interval of 0.630 μm. Dendritic spines were imaged with a Plan-Apochromat 100×/1.4 oil DIC objective, resolution of 34.2 pixels/μm and Z-stack interval of 0.258 μm. Sholl analysis was performed on 2D maximal intensity images acquired from Z-stacks, using the Fiji ImageJ plugin. The number of intersections was estimated with 10 μm bins, and values per cell were used for statistical analysis. For spine density analysis, three distant dendrites were imaged per cell. Spine density was evaluated on the primary dendrites for P7 and on the secondary for P21 and adult animals. Values were plotted to a density per 20 μm long section of the dendrite. Actual spine detection was done using the Fiji ImageJ plugin to quantify spines in a Z-stack image. Verification of the spine detection was done manually. The values for individual dendrites were averaged per cell. Values of spine density per cell were used for statistical analysis.

### Acute Brain Slice Preparation

For electrophysiological recordings and morphology analysis, postnatal day (P7), P21 and adult (6 weeks to 3 months old) animals were used. Adult animals were deeply anesthetized using halothane before sacrifice by decapitation. Brains were rapidly removed and placed in ice-cold cutting solution of the following composition (in mM): 140 C_5_H_14_ClNO (choline chloride), 2.5 KCl, 1.25 NaH_2_PO_4_, 7 MgCl_2_, 26 NaHCO_3_, 0.5 CaCl_2_, 10 D-glucose equilibrated with 95% O_2_-5% CO_2_ mixture. For recording NMDA mEPSCs, MgCl_2_ was excluded and 1.25 mM of kynurenic acid was added. Depending on the experimental purpose, coronal, parasagittal, or horizontal (tilted at 30°) cortico-striatal slices were prepared on a vibratome (Leica VT1000S). Slice thickness varied depending on the age of the animals (300 μm for P7–P21, 220 μm for adults). After preparation, slices were allowed to recover for 1 h at 34°C in artificial CSF with elevated Mg^2+^ concentration (unless NMDA-mEPSCs were being recorded; composition in mM: 127 NaCl, 2.5 KCl, 1.25 NaH_2_PO_4_, 3 MgCl_2_, 26 NaHCO_3_, 2 CaCl_2_, 15 D-glucose). All recordings were performed on MSNs located in the dorsal part of the striatum.

### Electrophysiology

During recordings, slices were perfused with aCSF (composition in mM: 127 NaCl, 2.5 KCl, 1.25 NaH_2_PO_4_, 1 MgCl_2_, 26 NaHCO_3_, 2 CaCl_2_, 15 D-glucose, 95% O_2_ – 5% CO_2_) at a rate of 1–2 ml/min. To record NMDA-mEPSCs, MgCl_2_ was excluded and 25 μM of D-Serine together with 1 μM of strychnine were added to aCSF. All recordings were made at 32°C. Whole-cell patch-clamp borosilicate-glass pipets (Hilgenberg) were obtained with a vertical two-stage puller (PIP 5; HEKA) and had a resistance typically between 5 and 7 MΩ. Current- and voltage-clamp recordings were obtained with an EPC-10 amplifier (HEKA Elektronik, Germany) controlled with PatchMaster (HEKA) software. Online filtering was achieved using the Bessel 2.9 kHz filtering with a sampling interval of 20 kHz. Analysis of the passive membrane parameters was done using a voltage-step protocol as described by D’Angelo et al. (1995).

#### Evoked Glycinergic Currents

Coronal cortico-striatal slices were used for recording evoked glycinergic currents from MSNs of C57Bl/6 mice of two ages (P7 and adults) and GlyRα2KO P5–8 mice. Recordings were done in whole-cell voltage-clamp (-80 mV holding potential) configuration, using CsCl-based internal solution (in mM): 120 CsCl, 0.022 CaCl_2_, 4 MgCl_2_, 10 HEPES, 0.1 EGTA, 5 phosphocreatine, 4 MgATP, 0.5 Na_2_GTP, 5 lidocaine N-ethyl chloride and 0.5% biocytin; pH 7.2. 10 μM GABA_A_ receptor blocker gabazine (Sigma-Aldrich), 10 μM AMPA receptor blocker CNQX, 0.1 μM nicotinic ACh receptor blocker DHβE, 5 μM NMDA receptor blocker L-689,560 and 1 μM voltage-gated sodium channel blocker TTX (other substances: Tocris) were present in aCSF. In case of GlyRα2KO mice, gabazine was omitted, and 3 mM GABA was used as additional control for barrel tube position and cell viability. Increasing concentrations of agonists or KCl (positive control) were applied using a fast-perfusion system (SF-77B, Warner Instruments) for a duration of 10 s.

#### Synaptic and Tonic Glycinergic Currents

The presence or absence of synaptic glycinergic currents was assessed by measuring mIPSCs in the presence of 10 μM gabazine (with CsCl-based internal solution and inhibitors of AMPA, NMDA receptors, and Na channels, as described below). The presence or absence of tonic glycinergic currents was evaluated by a change in holding current of the cell, patched with CsCl-based internal solution and held at -80 mV without any inhibitors, upon application of 1 μM strychnine. After the baseline period of 1 min, strychnine was applied using a fast-perfusion system, and the holding current was recorded for another 5 min. Mean values of holding current during the first and sixth min of the recording were measured.

#### Spontaneous Activity and Excitability

Spontaneous activity and intrinsic excitability of MSNs were evaluated using GlyRα2KO and WT neonatal mice (P6–7). Cells were recorded in perforated-patch or whole-cell current-clamp configurations. In these experiments, we used 30° tilted horizontal slices for preserving cortico-striatal connections (Jiang and North, 1991). For perforated patches, the pipette solution was composed of (in mM): 125 KMeSO_3_, 13 KCl, 10 HEPES, 140 μg/ml gramicidin. Measurements started after evaluating the access resistance in voltage-clamp mode by use of 10 mV-steps in holding potential starting from -70 mV (Akaike, 1996). RMP and spontaneous firing were recorded in current clamp configuration without any background current injected. For the whole-cell current-clamp configuration, the pipette solution was composed of (in mM): 116 K-gluconate, 15 KCl, 5 NaCl, 10 HEPES, 4 MgATP, and 0.5 Na_2_GTP. Membrane potential was recorded for 5 min without any background current injection. For evaluating of the active membrane properties and intrinsic excitability, cells were held at -70 mV, and depolarizing current steps with 10 pA increments were applied, starting from the background current. Individual action potential (AP) analysis was done as described by Chand et al. (2015). Briefly, for individual AP analysis, the first and last AP spike from the train were excluded (generated at 20 pA current injected from rheobase). Average phase plane plots (dV/dt vs. V) were generated from these traces. The slope and rise of the AP was measured by the linear fit to the phase plane plot. Spike width was measured at the midpoint between voltage threshold and maximum voltage.

#### Inward Rectifying Potassium Currents and Depolarization-Activated Potassium Currents

Recordings were done in the presence of 1 μM TTX. Both hyperpolarizing and depolarizing experiments were recorded in whole-cell voltage-clamp mode. Pipette solution was composed of (in mM): 116 K-gluconate, 15 KCl, 5 NaCl, 10 HEPES, 4 MgATP, and 0.5 Na_2_GTP. Steps of a 10 mV (for 1 s) were used in a range of -150 mV to 30 mV (from -70 mV baseline holding potential). Currents were measured as the average amplitude from baseline during the last 50 ms of each step. Currents were further normalized for cell capacitance which was measured by a voltage-step protocol as described by D’Angelo et al. (1995).

#### Miniature Excitatory and Inhibitory Postsynaptic Currents (mEPSCs and mIPSCs)

AMPA-mEPSCs, NMDA-mEPSCs, and mIPSCs were recorded in MSNs of GlyR2αKO and WT mice in a whole cell voltage clamp configuration at -80 mV. For mEPSCs, intracellular solution contained (in mM): 115 KMeSO_3_, 7 KCl, 0.022 CaCl_2_, 4 MgCl_2_, 10 HEPES, 0.1 EGTA, 5 phosphocreatine, 4 MgATP, 0.5 Na_2_GTP, and 0.5 % biocytin; pH 7.2. AMPA-mEPSCs were recorded in the presence of 10 μM gabazine, 0.1 μM DHβE, 5 μM L-689,560 and 1 μM TTX. For NMDA-mEPSCs, L-689,560 was excluded and 10 μM of CNQX was added to modified ACSF. For mIPSCs, we used a high chloride intracellular solution, the same as for recordings of evoked glycinergic currents. For mIPSC recording, gabazine was omitted and 10 μM CNQX was added into aCSF. Data were analyzed using the MiniAnalysis program (Synaptosoft).

#### Peak-Scaled Non-stationary Fluctuation Analysis (NSFA)

Non-stationary fluctuation analysis (NSFA) was performed from an ensemble of mEPSCs at a holding potential of -80 mV. Events were carefully selected for analysis by visual inspection based on the following criteria: fast rise time to allow for precise alignment, stable baseline, and exponential decay. To estimate the “open probability at peak” and the “number of channels at peak” of AMPARs the MiniAnalysis program (Synaptosoft) was used (Hartveit and Veruki, 2007; Lambot et al., 2016). An averaged mEPSC event was constructed aligning the individual events by their point of maximal rise. The average response waveform was scaled to the peak of individual responses and the variance of the fluctuation of the decay around the mean was calculated. The variance, in 100 bins of equal decrement, was plotted against the mean current amplitude of the decay. The parabolic relationship was fitted by following equation:

σ2(I) =iI−I^2/N+b

where i was the mean single-channel AMPAR current, I the mean current, N the number of channels activated at the peak, and b the baseline variance. Parameter i was estimated as the slope of the linear fit of the first portion of the parabola because the equation becomes linear when AMPAR open probability gets close to zero. The number of open channels at the peak was calculated by dividing the average mEPSCs amplitude by the unitary current i.

#### Evoked Excitatory Postsynaptic Currents (eEPSCs)

Glutamatergic responses in MSNs of GlyRα2KO and WT mice were evoked by electrical stimulation of corpus callosum by a concentric electrode. Recordings were made from parasagittal slices. Stimulation intensity was selected based upon an input – output protocol and set to the intensity that evoked eEPSCs with an amplitude of 50% of the maximum eEPSCs. For every part of the input – output protocol, 10–20 sweeps were recorded with 30 s interval time and then averaged during subsequent analysis. Ten micromolars gabazine (Sigma-Aldrich) and 1 μM CGP 35348 (Tocris) were added to the aCSF to block GABA_A_ and GABA_B_ receptors, respectively. For the strychnine condition, 1 μM strychnine (Sigma-Aldrich) was additionally added to the recovery aCSF and aCSF. Intracellular solution used was as follows (in mM): 110 CsMeSO_3_, 7 KCl, 0.022 CaCl_2_, 4 MgCl_2_, 10 HEPES, 0.1 EGTA, 5 phosphocreatine, 4 MgATP, 0.5 Na_2_GTP, 5 lidocaine N-ethyl chloride, and 0.5% biocytin; pH 7.2. The paired-pulse ratio was determined after giving two stimulations with 50 ms-interval, and dividing the amplitude of the second peak by the amplitude of the first peak. AMPA/NMDA ratios were estimated by measuring eEPSCs at different holding potentials. Glutamatergic currents were recorded at -70 mV and +40 mV; after that 5 μM NMDA receptor blocker L-689,560 was added to aCSF to isolate AMPAR current at +40 mV. NMDAR currents were calculated by subtracting the response recorded in the presence of L-689,560 at +40 mV, from the response recorded at +40 mV without this inhibitor. The AMPA/NMDA ratio was calculated by division of the amplitude of AMPAR-mediated response at -70 mV by the amplitude of NMDAR-mediated response.

### Behavioral Tests

#### Ultrasonic Vocalizations (USVs)

Ultrasonic vocalizations (USVs) were evaluated in WT and GlyRα2KO male neonatal pups at P4, P6, and P8. On each day of testing, pups were removed from the nest and placed in a clean empty plastic container located inside a sound-attenuating Styrofoam box. Room temperature was maintained at 22 ± 1°C. Recordings were performed using an Avisoft UltraSoundGate condenser microphone capsule CM16 with frequency range 10–180 kHz (Avisoft Bioacoustics, Germany), placed through a hole in the Styrofoam box approximately 20 cm above the pup. Vocalizations were recorded for 3 min with a sampling rate of 250 kHz and 16 bit format using Avisoft-RECORDER software V3.2. After testing, pups were weighed and returned to the nest. After testing on P4, pups were also individually identified with a paw tattoo. Acoustic analysis of audio files was performed in Avisoft SASLab Pro V4.4. Spectrograms were generated with a Fourier transformation length of 1024 and time window overlap of 75% (100% frame, Hamming window). A high pass filter at a cut-off frequency of 15 kHz was applied to remove background noise outside the relevant frequency band. Call detection was by an automatic threshold-based algorithm and a hold-time mechanism (hold time 20 ms). An experienced user blinded to genotype checked accuracy of call detection to ensure concordance between automated and observational detection. Parameters extracted for each time point included mean number of calls/min, mean duration of calls, and quantitative analyses of sound frequencies.

#### Neonatal Righting Reflex

Neonatal righting reflex was assessed in P5, P6, and P7 WT and GlyRα2KO male littermates. Briefly, pups were placed in a horizontal recumbent position on a cleaned flat surface free of obstacles. The time pups needed to right onto all fours was measured. If pups did not right after 1 min, a time of 60 s was recorded, and the test was stopped. Each pup was evaluated three times per experimental day.

### RNA and Protein Isolation

Animals at different developmental stages were anesthetized and decapitated for brain extraction. Brains slices of 300 μm were prepared on a vibratome (Leica VT1000S) in RNase-free 0.01 M PBS solution. Dorsal striatum was dissected for RNA extraction. For qPCR and Western blotting, RNA and proteins were isolated simultaneously by guanidinium thiocyanate-phenol-chloroform (AGPC) method using QIAzol lysis reagent (Qiagen, Germany). Briefly, samples were homogenized in QIAzol and the RNA was separated to an upper phase whilst the proteins remained in the lower phase. The upper phase was collected and further decontaminated by a second chloroform step. Subsequently, the RNA and proteins were precipitated separately and washed according to the QIAzol protocol. The quality and quantity of the RNA was accessed using the NanoDrop 1000 (Thermo Fisher Scientific). Synthesis of cDNA was performed by High Capacity cDNA Reverse Transcription Kit (Applied Biosystems^TM^, Thermo Fisher Scientific, Belgium). The protein pellet was dissolved in 100 μl 1% sodium dodecyl sulphate (SDS) supplemented with Pierce^TM^ Protease Inhibitor Mini Tablets (Thermo Fisher Scientific) and stored at -20°C. The protein concentration was determined by bicinchoninic acid assay (Pierce BCA protein assay kit, Thermo Fisher Scientific).

### RT-QPCR and Analysis

cDNA amplification was performed using Fast SYBR green master mix (Applied Biosystems) containing 3 mM forward and 3 mM reverse oligonucleotide primers (**Table 1**), RNAse-free water, and 12.5 ng cDNA template in a total reaction volume of 10 μl. Universal cycling conditions were used (95°C for 20 s, 40 cycles of 95°C for 3 s, and 60°C for 30 s) (Jiang and North, 1991). Specificity of PCR products was determined using melting curve analysis (StepOne Software V2.3) and molecular weight analysis (2% agarose gel) following the amplification protocol. Efficiency of PCR products was evaluated using standard curves of pooled samples. Data were normalized against the most stable reference genes as determined by qBasePlus software (Biogazelle). Relative quantification of gene expression was performed using the comparative 2^-ΔΔ*Ct*^ method (Pfaffl, 2001). Expression levels were converted to fold change values for gene expression and compared to the averaged wild-type value of the youngest tissue samples tested for that specific gene, which was set to one.

**Table 1:** Sequence and supplier information of primers used.

Gene	Forward primer	Reverse primer
*RPL13A*	5′-CTGGTACTTCC ACCCG ACCTC-3′	5′-GGATCCCTCCACCCTAT GACA-3′
*PGK1*	5′-GAAGGGAAGGGAAA AGATGC-3′	5′-GCTATGGGCTCGGT GTGC-3′

***Gene***	**Supplier**	**Cat. No.**

*GLRA1*	Qiagen	QT00172221
*GLRA2*	Qiagen	QT00132020
*GLRA3*	Qiagen	QT01657943
*GLRB*	Qiagen	QT00162911


### Western Blotting

Isolated and dissolved proteins were mixed with sample buffer (5:1, v/v) containing 300 mM TrisHCl (pH 6.8), 6% SDS, 30% glycerol, 15% β-mercaptoethanol, and 0.01% bromophenol blue. Samples containing 20 μg protein were separated by 10% SDS polyacrylamide gel electrophoresis (SDS-PAGE) in Mini-PROTEAN systems (Bio-Rad) and transferred onto polyvinylidene fluoride (PVDF) membranes. Membranes were blocked with 5% BSA in Tris-buffered saline containing 0.1% Tween-20 (TBS-T) for 1 h at room temperature. The blots were probed with primary antibody, rabbit anti-PSD95 (1:1000; ab18258, Abcam) and guinea-pig anti-VGluT1 (1:1000 ab5905, Millipore) in blocking solution overnight at 4°C. β-Actin was used as a reference. The membranes were incubated for 1 h at RT with subsequent HRP-conjugated secondary antibodies in TBS-T. Signal was evoked using Pierce ECL 2 Substrate (Thermo Fisher Scientific). Bands were visualized by chemiluminescent imaging (ImageQuant^TM^ LAS 4000 mini). Analyses were performed utilizing 16-bit scaled intensity measurements of target, reference and background areas in FIJI ImageJ (Schindelin et al., 2012). Results were compared to the averaged value of P7 WT samples, which was set to one.

### Data Analysis and Statistics

Curve fitting and data analyses were performed with Igor Pro 6.0 (WaveMetrics) and GraphPad Prism 7. Histogram and box plots were used for graphic representation. On the histograms, mean with SEM are shown. On the box plot, the central line represents the median, the edges represent the interquartile ranges, and the whiskers represent the overall distribution. The D’Agostino-Pearson omnibus normality test was used to determine a normal distribution. Pairwise comparisons were performed on normal distribution using unpaired or paired *t*-test (unless specifically otherwise mentioned), as appropriate; non-normal distributions were analyzed by a Mann–Whitney *U* test. Some data were analyzed by a one-way repeated-measures ANOVA followed by a Tukey’s multiple comparison *post hoc* test or by a two-way repeated-measures ANOVA followed by Bonferroni multiple comparisons *post hoc* test, depending on the experimental design. The level of significance was established as follows: ^∗^*p* < 0.05, ^∗∗^*p* < 0.01, and ^∗∗∗^*p* < 0.001.

## Results

### The GlyR α2 Subunit in MSNs Is Downregulated During Postnatal Development

**FIGURE 1:**
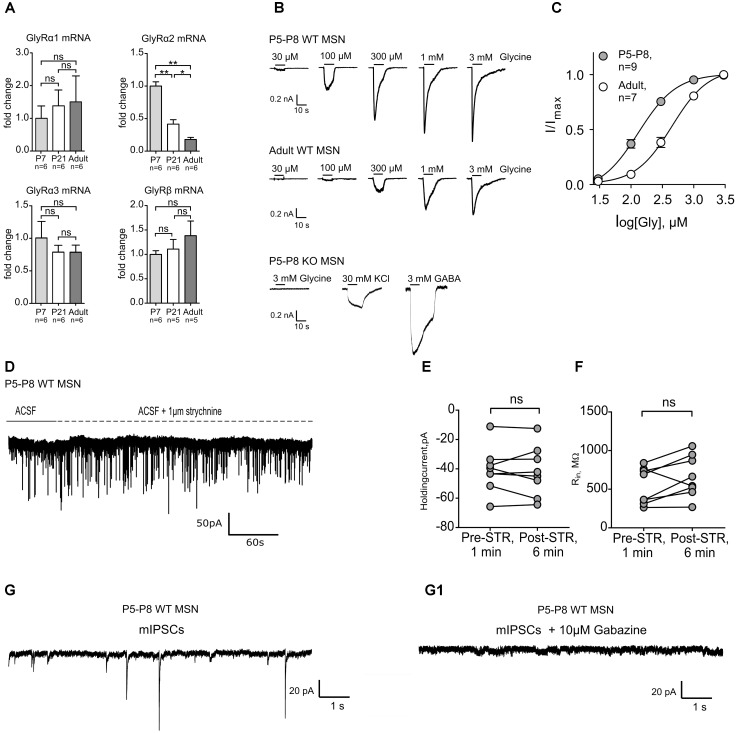
Transcriptional and functional comparison of GlyRs at different developmental stages. The GlyR α2 subunit mRNA is developmentally down-regulated which corresponds to the lower current density (pA/pF) of evoked glycinergic currents in adult dorsal striatum MSNs. **(A)** qPCR results from dorsal striatum show a stark decrease in the transcription of the GlyR α2 subunit gene (^∗^*p* < 0.05; ^∗∗^*p* < 0.01). Data are normalized to the expression at P7 and presented as mean ± SEM. GlyR α1, α3, and GlyR β subunit mRNAs did not show a developmental up- or down-regulation. **(B)** Representative dose-response traces from glycine-evoked currents in whole cell. In the P5-P8 GlyRα2KO mice no glycinergic currents were measured in dorsal striatum MSNs (*n* = 7). **(C)** Relative dose-response curves from neonate (P5-P8) and adult WT MSNs to glycine. **(D)** Representative trace of a P5-P8 MSN clamped at –80 mV with application of ACSF (1 min) and ACSF + STR (5 min). **(E,F)** values of the holding current (pA) and input resistance (MΩ) measured after 1 min of ACSF (Pre-STR) or 5 min after ACSF+strychnine (Post-STR) application in WT P5-P8 MSNs (*n* = 8). **(G,G1)** Representative traces of phasic GABAergic mIPSCs and **(G1)** phasic current in the presence of gabazine. mIPSCs were isolated by application of inhibitors, as described in the Section “Materials and Methods.”

We have shown previously that MSNs of adult mice express functional GlyRs with α2 being the main agonist-binding subunit (Molchanova et al., 2018). To assess the developmental role of GlyRs in the striatum, the expression pattern of the different GlyR subunits was first identified in the developing DS. Quantitative real-time PCR on microdissected DS of mice at different ages showed that the expression of *Glra2* is developmentally down-regulated during maturation (**Figure 1A**). However, *Glra1*, *Glra3*, and *Glrb* did not exhibit a developmental change in their expression levels (**Figure 1A**) and the overall expression of *Glra1* and *Glra3* was around 20-times lower than for *Glra2* (**Supplementary Figures 1A,B**). For *Glra4*, expression remained undetected and was consequently not included in the results. In fact, *Glra1* and *Glra3* mRNAs were barely detectable and this finding was confirmed by functional analysis. In both neonatal (P5–8) and adult WT MSNs, glycinergic currents were measured in whole-cell patch-clamp mode, but none of the neonatal MSNs showed a glycinergic response in GlyRα2KO (KO) mice (**Figure 1B**). This implies that the GlyR α2 subunit is the only ligand binding subunit to be present at functionally relevant expression levels in developing MSNs, similar to adult ones (Molchanova et al., 2018). The maximum current density was also significantly higher in neonatal versus adult MSNs, which might suggest that the function of this glycinergic current changes during development (**Table 2**). The relative dose-response curve shows a rightward shift for the adult MSN population as compared to neonatal MSNs (**Figure 1C**). This correlates with a lower effective concentration (EC_50_) in the neonatal MSNs for glycine as compared to the adult MSNs (**Table 2**). Together, these data indicate that the GlyR α2 subunit is the only agonist-binding subunit present at all stages of development, and that it has a higher receptor density and affinity in developing MSNs as compared to the adult MSNs, highlighting its importance for developing striatal MSN population.

**Table 2:** Characteristics of evoked glycinergic currents in neonatal and adult MSNs (mean ± SEM).

	WT MSNs, P5-P8	WT MSNs, adult	*P*-value
EC_50_ (μM)	139.24 ± 16.87	470.44 ± 67.15	0.0002
Hill coefficient	1.52 ± 0.10	1.63 ± 0.08	0.2509
Max. current density (pA/pF)	22.75 ± 3.69	5.54 ± 1.60	0.0021
*N*	9	7	


### At P7, No Glycinergic Synaptic or Extrasynaptic Currents Are Present in Neostriatal MSNs

While we established the presence and characteristics of the evoked glycinergic currents in neonatal MSNs, their physiological function remains unidentified. Tonic and phasic glycinergic signaling depends on receptor localization, i.e., extrasynaptic and synaptic, respectively. In adult dorsal MSNs, endogenous glycinergic currents have been found to work via tonic receptor activation (Molchanova et al., 2018). However, considering this fact, it is well known that these processes are often developmentally regulated and thus age-dependent (Valtcheva et al., 2017). Using a high-chloride filling solution in the whole-cell configuration we tested for the presence of both types of glycinergic signaling. In neonatal MSNs, at P6–P8, no significant change in holding current was detected in WT animals upon strychnine application (**Figures 1D–F**; IHold Pre-STR: -40.74 ± 5.497 pA vs. Post-STR: -41.68 ± 6.034 pA). The same outcome was observed in MSNs of the GlyRα2KO animals (**Supplementary Figures 1C,D**). Furthermore, when all ionotropic glutamate and GABA receptors where pharmacologically blocked, in the presence of TTX, no currents could be detected indicating of the absence of functional synaptic GlyRs (**Figures 1G,G1**). This strongly suggests that there is no tonic or phasic glycinergic signaling in neonatal dorsal MSNs.

### Spontaneous and Evoked Action Potential Firing Is Impaired in Neonatal GlyRα2KO Mice

**FIGURE 2:**
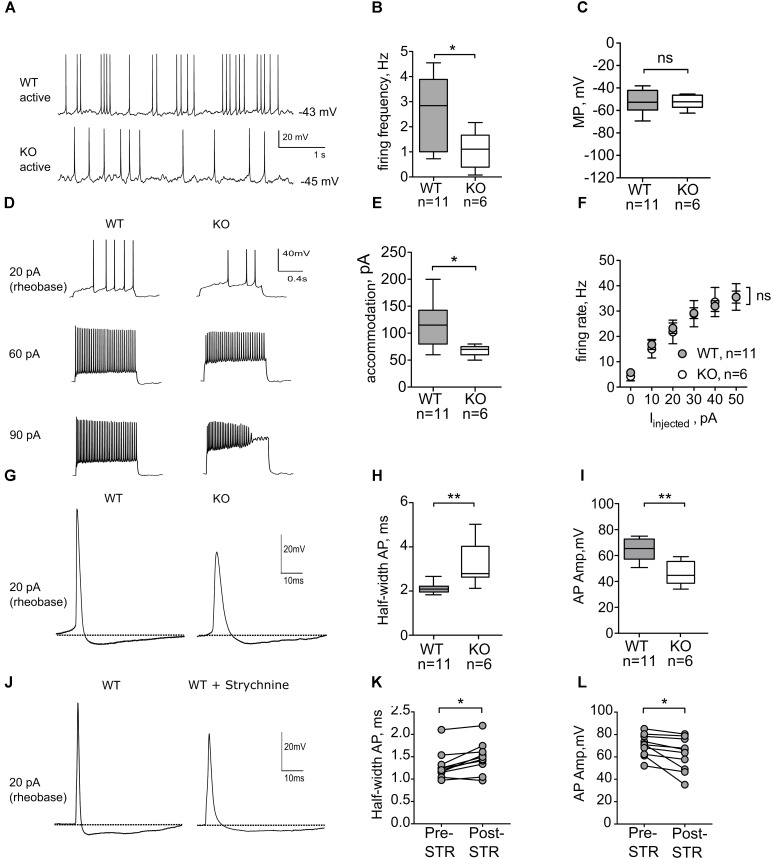
Active neonatal MSNs from GlyRα2KO mice (P6-7) have slower rates of spontaneous firing and altered properties of evoked action potentials. **(A)** Representative traces from spontaneous activity in WT and KO MSNs. **(B)** Firing frequency of spontaneous action potentials is significantly reduced in the KO MSNs. **(C)** Membrane potential of active WT and KO MSNs. **(D)** Representative traces showing evoked action potentials in active MSNs in WT and KO dorsal striatum. **(E)** Injected current, needed to evoke accommodation in active WT and KO MSNs. **(F)** Action potential firing rate as a function of injected current, starting from rheobase. **(G)** Example traces of individual action potential, recorded in WT and KO MSNs. **(H,I)** Action potential half-width (ms) and amplitude (mV) of individual action potentials from WT and KO MSNs. **(J)** Example traces of individual action potentials, recorded in WT MSNs before and after 5 min of 1 μM strychnine application. **(K,L)** Action potential half-width (ms) and amplitude (mV) of individual action potentials recorded in WT and KO MSNs after 1 μM strychnine application. Pooled data are presented as either mean ± SEM or box plot (^∗^*p* < 0.05, ^∗∗^*p* < 0.01).

Early postnatal spontaneous activity has been described as a mechanism underlying the development of neurons and synapses for many brain structures, including dorsal striatum (Kerschensteiner, 2014). Since agonist-gated chloride channels, such as GABA_A_Rs and GlyRs, play a major role in initiation and propagation of neonatal activity (Ben-Ari, 2002), we evaluated the role of GlyRs in action potential firing of MSNs in P6–7 mice. Spontaneous and evoked action potential firing was recorded in wild-type and GlyRα2KO MSNs using the perforated patch-clamp method in order to minimally influence the intracellular environment (Vogt, 2015). In the dorsal striatum of WT animals, 11 out of 35 cells (“active” MSNs, 31%) fired spontaneous action potentials (**Figure 2A**), when recorded in current-clamp mode without any background current injection. These spontaneously active cells seem to be less developed, than silent cells, based on the firing frequency of the APs, evoked by depolarizing steps, and RMP (**Supplementary Figure 2**). Spontaneously active MSNs displayed an irregular firing pattern, and no bursting activity was observed. In GlyRα2KOs, a similar fraction of MSNs was spontaneously active (**Figure 2A**; 6 out of 17 cells, 35%; *p* = 0.8 by Fisher’s test). Quantification of the spontaneous firing frequency (**Figure 2B**) indicated that the frequency of action potentials was significantly lower in the GlyRα2KOs (1.08 ± 0.31 Hz) as compared to the WT MSNs (2.37 ± 0.43 Hz; *p* < 0.05). This change in firing frequency was not accompanied by a significant change in the membrane potential (**Figure 2C**), and passive membrane properties, such as input resistance and cell capacitance remain unaffected (**Table 3**). To rule out NMDA mediated component of spontaneous activity, we recorded NMDA-mEPSCs. At this developmental stage, NMDA-receptors are of the NR2C/D type which are known to have longer decay time and are crucial for synapse-driven calcium events in immature MSNs (Dehorter et al., 2011). No significant differences were found in NMDA-mEPSCs in WT versus GlyRα2KOs for either frequency (WT: 0.26 ± 0.02 Hz, KO: 0.22 ± 0.01 Hz) or amplitude (WT: 16.45 ± 0.75 pA, KO: 15.97 ± 0.70 pA) of the events (**Supplementary Figure 3**).

**Table 3:** Passive membrane properties and intrinsic excitability parameters of active neonatal MSNs from wild-type and GlyRα2KO animals (mean ± SEM).

	Active MSNs, WT P6-7	Active MSNs, GlyRα2KO P6-7	*P*-value
Input resistance (MΩ)	1555 ± 296.8	1975 ± 496.1	0.403
Capacitance (Cm)	89.55 ± 17.43	53.88 ± 8.322	0.385
Rheobase (pA)	24 ± 5.812	18.33 ± 3.073	0.821
Threshold (Vm)	-40.05 ± 3.385	-39.37 ± 1.679	0.727
Ihold to -70 mV (pA)	-17.55 ± 4.764	-13.83 ± 2.686	0.862
Rate of AP rise (mV/ms)	150.80 ± 17.30	62.72 ± 10.94	0.002
Rate of AP decay (mV/ms)	30.76 ± 1.88	15.38 ± 2.52	0.001
Fast AHP, half-width (ms)	25.86 ± 1.951	23.39 ± 2.195	0.097
Fast AHP, amplitude (mV)	5.468 ± 0.215	6.109 ± 0.387	0.802
*N*	11	6	


To further evaluate the intrinsic excitability of spontaneously active neonatal DS MSNs, current was injected intrasomatically in the current-clamp mode, starting from membrane potential values of -70 mV (**Figure 2D**). In both WT and GlyRα2KO MSNs, first APs were evoked at similar threshold and rheobase values (**Table 3**). Upon increase of the values of injected current, WT and GlyRα2KO cells fired APs with similar frequencies (**Figure 2F**). However, in GlyRα2KO MSNs, APs started to accommodate at significantly lower values compared to wild-type MSNs (**Figure 2E**, 68 ± 4.90 pA in KOs vs. 117 ± 14.07 pA in WTs; *p* < 0.05).

**FIGURE 3:**
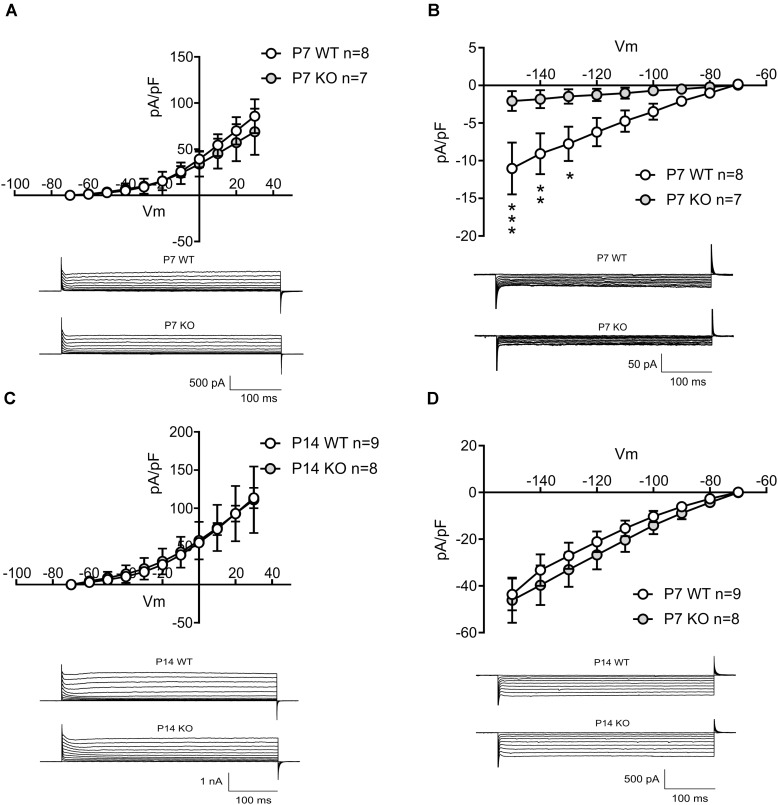
Evaluating the outward and inward potassium currents in P7 active MSNs and P14 silent MSNs in WT and KO mice. **(A)** Current density plotted for depolarizing membrane potentials in P7 active MSNs in WT and KO, and example traces of outward potassium currents recorded in P7 active MSNs in WT and KO mice. **(B)** Current density plotted for hyperpolarizing membrane potentials in P7 active MSNs in WT and KO, and example traces of inward potassium currents recorded in P7 active MSNs in WT and KO mice. (*n*_WT_ = 8; *n*_KO_ = 7). **(C)** Current density plotted for depolarizing membrane potentials in P14 MSNs in WT and KO, and example traces of outward potassium currents recorded in P14 MSNs in WT and KO mice. **(D)** Current density plotted for hyperpolarizing membrane potentials in P14 MSNs in WT and KO, and example traces of inward potassium currents recorded in P14 MSNs in WT and KO mice. (*n*_WT_ = 9; *n*_KO_ = 8). Data are presented as mean ± SEM (^∗^*p* < 0.05, ^∗∗^*p* < 0.01, ^∗∗∗^*p* < 0.001).

To explain the decreased spontaneous firing frequency and accommodation current in GlyRα2KOs, an individual AP analysis was performed (**Figure 2G**). Both the amplitude and duration of APs in spontaneously active GlyRα2KO cells were severely affected. In GlyRα2KO DS MSNs, the AP half-width is higher (**Figure 2H**, 3.20 ± 0.43 ms) as compared to WT DS MSNs (2.05 ± 0.08 ms; *p* < 0.05). This was accompanied by significant changes in the rise and decay rates (**Table 3**). The amplitude of the APs differed significantly when comparing “active” KO and WT cells (**Figure 2I**). No significant changes were found in the after-hyperpolarization parameters (**Table 3**). We were able to replicate these results when we acutely blocked the GlyR α2 subunit with strychnine: using a modified intracellular solution, we recorded in whole-cell current clamp configuration and evoked action potentials (at rheobase + 20 pA) in spontaneously active MSNs. In recordings from the same WT cells, the action potential broadened after 5 min application of strychnine (**Figures 2J–K**, Pre-STR: 1.30 ± 0.10 ms, Post-STR: 1.48 ± 0.11 ms; *p* < 0.05). Similarly, a significant decrease was also found for the amplitude of the action potential (**Figures 2J,L**, Pre-STR: 70.33 ± 3.14 mV, Post-STR: 62.14 ± 4.73 mV; *p* < 0.05). We furthermore looked at inward rectifying currents and potassium currents activated by depolarization in P7 “active” WT and KO MSNs and P14 WT and KO MSNs (no spontaneous APs were seen in P14 MSNs). At P7 we found significantly lower inward rectifying currents (**Figure 3B**) for the voltage steps -150, -140, and -130 mV (respectively, pA/pF; WT: -11.025 ± 3.43 KO: -2.068 ± 0.5; WT: -9.07 ± 2.72 KO: -1.833 ± 0.45; WT: -7.777 ± 2.72 KO: -1.470 ± 0.36). This was, however, not observed in the more developed P14 MSNs (**Figure 3D**) indicating a transient effect at P7. This reduced functioning of inward rectifying current might be a consequence of either delayed development of the MSNs, or loss of GlyR-mediated modulation of some inward-rectifying potassium channels (Dehorter et al., 2011). No effects were observed on the voltage-gated potassium currents (**Figures 3A,C**).

These data indicate that GlyR α2 affects the spontaneous activity in neonatal MSNs and, when pharmacologically blocked or genetically ablated, cause the broadening of the AP waveform and reduced frequency of spontaneously occurring APs in “active” cells, without affecting the passive membrane properties or after-hyperpolarizing potentials. Probably, the effect of GlyR α2 is mediated by some inward rectifying potassium channel. Interestingly, we did not find any difference in passive membrane properties and intrinsic excitability properties when comparing “silent” neonatal MSNs from WT and GlyRα2KO animals (**Supplementary Table 1**). Since “silent” cells seem to be functionally more developed, than “active” cells, it appears that the impact of GlyRs on the firing properties of MSNs is only present at certain developmental stages and disappears with maturation of the cell. However, the role of GlyRs in AP firing of neonatal MSNs may have dramatic consequences on the density of synaptic connections in adult animals.

### Functional Glutamatergic Innervation Is Reduced in GlyRα2KO Animals

**FIGURE 4:**
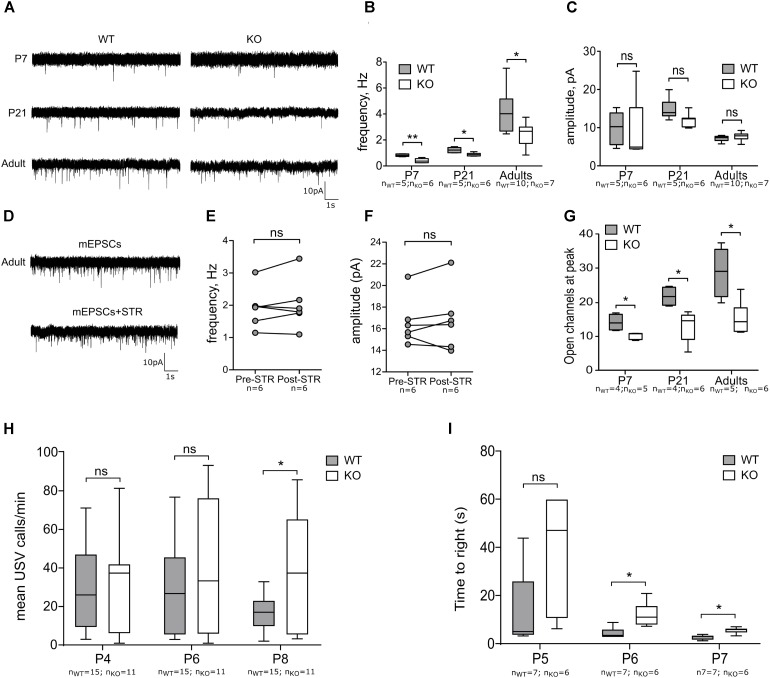
Functional glutamatergic innervation of MSNs is decreased in GlyRα2KO mice. **(A)** Example traces of mEPSCs in WT and KO MSNs of different ages. **(B,C)** Frequency and amplitude of mEPSCs in WT and KO MSNs of different ages. **(D)** Example traces of mEPSCs, recorded from MSNs of wild-type adult mice in control condition and after 1 μM strychnine application. **(E,F)** Frequency and amplitude of mEPSCs before and after strychnine application. **(G)** Peak-scaled non-stationary fluctuation analysis of the mEPSCs, recorded from MSNs of WT and KO animals. **(H)** Mean number of ultrasonic vocalizations emitted per min in male WT and KO pups at P4, P6, and P8. **(I)** Graph constructed from average time pups used to right on all fours. Data are presented as either box plot (whiskers indicate variability from minimum to maximum) or individual experimental values; ^∗^*p* < 0.05, ^∗∗^*p* < 0.01.

Proper glutamatergic innervation is essential for the MSNs to overcome the strong inhibitory inputs received from other MSNs and interneurons and thus convey cortical and thalamic signals to downstream motor nuclei (Surmeier et al., 2007). Both genetic factors (Chen et al., 2016) and intrinsic activity (Huupponen et al., 2013) fine tune the development of glutamatergic synapses and here we evaluated the contribution of GlyR α2 in functional maturation of the glutamatergic innervation of MSNs. First, we recorded AMPA-mEPSCs at different developmental stages (P7, P14 and adults) (**Figure 4A**). In all developmental stages, we observed a reduction of the frequencies of mEPSCs (**Figure 4B**). The amplitude of these events was not affected (**Figure 4C**). This implies that number of functional glutamatergic synapses is smaller in the GlyRα2KO DS MSNs without affecting the NMDA (frequency and amplitude) component of the glutamatergic transmission (**Supplementary Figure 3**). GABAergic synaptic transmission was not affected by deletion of *Glra2*, since the properties of mIPSCs were unchanged in adult GlyRα2KO MSNs as compared to WT (**Supplementary Figure 4**). To rule out the acute contribution of a direct modulation of glutamatergic transmission by GlyRs, we evaluated the acute effect of GlyR antagonist strychnine (1 μM) on mEPSCs recorded from DS MSNs of WT adult mice. Application of strychnine did not cause any reduction in the frequency and amplitude of mEPSCs (**Figures 4D–F**). In addition, the single synaptic parameter of open channels at peak of the averaged mEPSC event was evaluated by non-stationary fluctuation analysis (**Figure 4G**). The number of open channels was reduced throughout development suggesting an impaired development of AMPA-mediated mEPSCs. In conclusion, these data demonstrate that the number of functional glutamatergic synapses onto GlyRα2KO MSNs is reduced from early postnatal development and persists into adulthood. Notably, this reduction is not due to a direct effect of glycinergic transmission because strychnine application was not able to reproduce this. To see if reduced glutamatergic input onto the MSNs would be seen in motoric changes we used the righting reflex test. Disturbed motor behavior was observed in postnatal right reflex, measured as the time to right on all four paws. This was significantly slower in GlyRα2KO pups starting at P6 (**Figure 4I**). In the mouse, the motor regions of the brain, including the striatum, are activated during vocal production (Arriaga et al., 2012) due to intrinsic MSN activity and glutamatergic connectivity originating from the cortex to the MSNs (Kim et al., 2012; Araujo et al., 2015). The rate of neonatal vocalization (mean calls/min) upon isolation from the nest is significantly increased at P8 in GlyRα2KO compared to WT controls, but not at earlier time points (**Figure 4H**). This suggests that the developmental decrease in vocalization that normally occurs after peak vocalization at P6–P7 (Scattoni et al., 2008; Ey et al., 2013; Araujo et al., 2015) is delayed in GlyRα2KO mice. To explain these findings, we investigated the pre- and post-synaptic molecular and morphological characteristics of synapses on MSNs in WT and GlyRα2KO mice.

### The Number of Glutamatergic Synaptic Contacts Is Not Affected by *Glra2* Deletion

**FIGURE 5:**
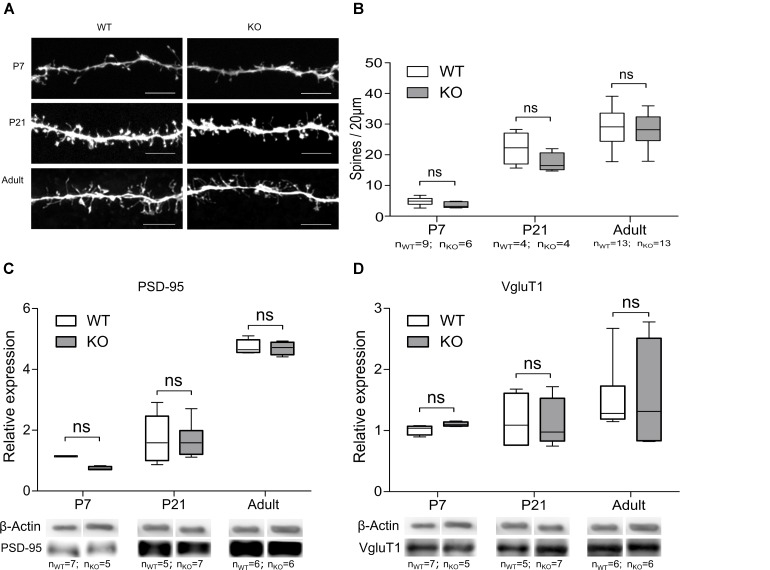
Deletion of the GlyR α2 subunit does not affect the number of glutamatergic synaptic contacts on MSNs. **(A)** Example images of MSN spines in WT and KO animals of different ages. Scale bar is 5 μm. **(B)** Quantification of spine density. **(C,D)** Representative Western blots and quantification of the band intensity of PSD-95 **(C)** and VGluT1 **(D)** in dorsal striatum of WT and KO animals of different ages. Data are presented as box plot (whiskers indicate variability from minimum to maximum).

Previous reports have demonstrated that the absence of the GlyR α2 subunit causes changes in synaptic density and neuronal ramifications in the cortex (Morelli et al., 2016). Similar processes might contribute to the observed reduction of functional glutamatergic inputs on MSNs in the dorsal striatum. We therefore performed an analysis of the morphology of the DS MSNs in WT and GlyRα2KO mice at different developmental stages. The morphology of the DS MSN dendritic tree was not affected in GlyRα2KO mice of different ages in comparison to WT mice (**Supplementary Figures 5A,B**). Furthermore, in P7, P21 and adult DS MSNs, no alterations of the spine density were seen for GlyRα2KO mice as compared to WT mice (**Figures 5A,B**).

Besides morphological changes, expression of synaptic proteins might also be an underlying mechanism for the observation of impaired glutamatergic network formation (Peca et al., 2011). The protein expression of the post-synaptic AMPAR and NMDAR anchoring protein PSD-95, and pre-synaptic corticostriatal vesicular glutamatergic transporter protein VGluT1 were evaluated (Berry and Nedivi, 2017; Fleischer et al., 2017). PSD-95 (**Figure 5C**) was expressed in equal amounts which is indicative of an unaltered number of glutamatergic synapses (Peca et al., 2011). The cortical glutamatergic afferents are also unlikely to be affected in the absence of the GlyR α2 subunit, as was evident from Western blot analysis of VGluT1 (**Figure 5D**). This suggested that the reduction in functional glutamatergic innervation in GlyRα2KO was unlikely to be a consequence of an impaired morphological development or spine generation of the DS MSNs at any developmental stage we investigated. A more in-depth analysis of the glutamatergic synapse in the dorsal striatal MSNs was therefore essential.

### Reduction of the Functional Connectivity May Be Due to Impaired Maturation of Silent Synapses

**FIGURE 6:**
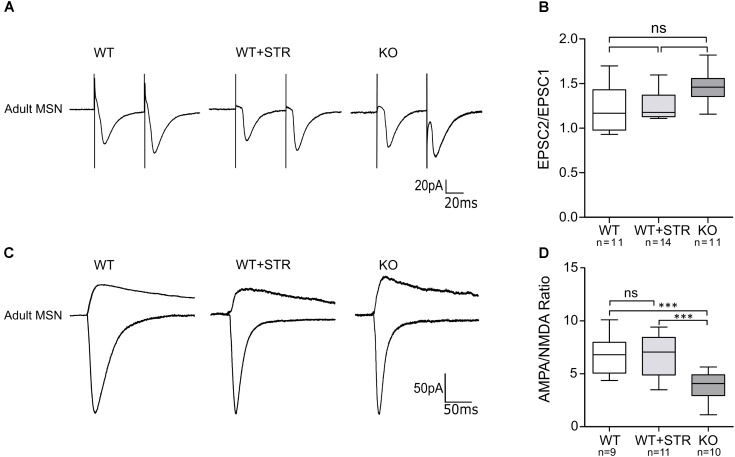
AMPA/NMDA ratio, but not paired pulse facilitation is reduced in MSNs of adult GlyRα2KO mice. Currents were evoked by stimulation through a concentric electrode placed at the corpus callosum. **(A)** Example traces of paired stimulation of AMPAR-mediated currents in WT MSNs, WT MSNs in the presence of 1 μM strychnine and in GlyRα2KO MSNs. Two stimuli were given with 50 μs interval. **(B)** Paired-pulse response ratio in control, strychnine-treated and knockout cells. **(C)** Example traces of AMPAR and NMDAR-mediated currents in MSNs of adult WT (control and strychnine-treated) and KO animals. AMPAR-mediated response was recorded at –70 mV and directed downward. NMDAR-mediated response is a result of subtraction of the response, recorded at +40 mV and the response in the presence of L-689,560. **(D)** Quantification of AMPA/NMDA ratio in MSNs of WT control, WT strychnine-treated and KO animals. Data are presented as box plot (whiskers indicate variability from minimum to maximum), ^∗∗∗^*p* < 0.001.

Reduction in the frequency of AMPA-mEPSCs in GlyRα2KO MSNs without any morphological changes might be the result of a pre- or postsynaptic defect. Developmental defects of the cortical afferent might lead to a reduced release probability of glutamatergic vesicles that might not be detected by measuring VGluT1 levels. Also, the existence of presynaptically located GlyRs cannot be ruled out. Postsynaptic defects might be the result of early changes in intrinsic activity which would manifest by changes in the AMPA/NMDA ratios. To evaluate these two possibilities, we recorded the paired-pulse ratio (PPR) of AMPAR-mediated evoked excitatory postsynaptic currents (eEPSCs) (**Figure 6A**) and measured the AMPA/NMDA eEPSC ratio (**Figure 6C**). No effect on the PPR was found in either the GlyRα2KO (PPR: 1.46 ± 0.05) mice or when the GlyRs were pharmacologically blocked (PPR WT: 1.23 ± 0.08; WT+STR: 1.26 ± 0.04) (**Figure 6B**). Interestingly, we saw a reduction in the AMPA/NMDA-ratio that was only present in GlyRα2KO mice (**Figure 6D**). No acute effects were detected when GlyRs were pharmacologically blocked (WT: 6.78 ± 0.60; WT+STR: 6.68 ± 0.59; KO: 3.84 ± 0.42). In summary, we can state that the post-synaptic reduction in AMPA/NMDA parameter is indicative of a synaptic maturation deficit in the GlyRα2KO, while the pre-synaptic vesicle release probability is unaffected by the *Glra2* knockout. This decrease in the AMPA/NMDA ratio may be a consequence of a reduction of AMPARs, as shown by non-stationary fluctuation analysis of mEPSCs, rather than an increase in NMDARs.

## Discussion

This work establishes a link for the first time between the GlyR α2 subunit and the development of glutamatergic synapses in MSNs of the dorsal striatum. In the mammalian nervous system, GlyR α2 is mainly present during early development, with expression attenuated or disappearing in adulthood (Young-Pearse et al., 2006). By examining the expression pattern of the different GlyR subunits and glycinergic current densities during the development of the striatum, we observed a high expression of *Glra2* in neonatal striatum and corresponding glycinergic current in striatal MSNs, which decreases during maturation. In adult murine striatum, GlyR α2 is the main ligand binding subunit (Molchanova et al., 2018), and we confirmed here that during postnatal development, there is no elevated expression of either GlyR α1 or GlyR α3 subunits. In the MSNs of neonatal GlyRα2KO mice, no glycine-evoked current was observed. Expression of *Glra1* and *Glra3* is still detectable at all ages examined, but significantly lower than that of *Glra2*. Since QPCR studies were performed in whole striatum, the interneuronal expression of Glra1 and 3 can contribute to detected mRNA levels (Sergeeva and Haas, 2001). We also observed a developmental shift in the EC_50_ and current density of evoked glycinergic responses. Considering the fact that the expression of the GlyR β subunit gene (*Glrb*) does not differ between neonatal and adult MSNs, we cannot attribute this effect to receptor composition (homomeric vs. heteromeric) (Wang et al., 2006). Alternatively, this might be a consequence of either cytoplasmic modulators that affect agonist binding capabilities of the GlyRs or the sensitivity might also be influenced by a higher receptor density in neonates which affects GlyRs inter-receptor interactions (Taleb and Betz, 1994; Fucile et al., 2000).

Taken together, our data indicate that GlyR α2 is the main agonist-binding subunit of GlyRs in striatal MSNs, and that the expression of *glra2* is developmentally regulated. However, the mechanism by which α2 subunit GlyRs mediate their actions in the neonatal MSNs seem to differ from their action in adult MSNs. In our recent study, it has been shown that GlyRs are extrasynaptic and control the RMP and excitability in adult MSNs (Molchanova et al., 2018). In neonatal MSNs, however, such tonic activity was not evident, which explains the absence of differences in RMP between GlyRα2KO MSNs and wild-type controls. This is similar to the development of striatal extrasynaptic GABA_A_ receptors, which do not show any tonic activity up until later postnatal ages (Valtcheva et al., 2017). When blocking all inhibitory receptors, excluding the GlyR, interestingly, we did not observe any synaptic inhibitory currents, neither in neonates nor in adults (Molchanova et al., 2018). This means that functionally active GlyRs are present in neonatal MSNs, but do not seem to be activated by classic synaptic release, or extrasynaptic spill-over of neurotransmitter.

Despite the absence of intrinsic glycinergic currents, α2 subunit GlyRs seem to regulate the excitability of neonatal MSNs. In early developmental stages, when sensory inputs are still immature, neurons spontaneously generate the electrical activity, which serves as a testing template for newly formed synaptic connections (Blankenship and Feller, 2010). These developmental stages are called “critical periods,” when neuronal networks are highly plastic and connections may change easily and form the most effective architecture to fulfill the adult-type behavioral requirements. The presence of early network activity has been shown in striatum before (Dehorter et al., 2011), and its importance regarding network formation we now also confirm. At the age of 6–7 days, roughly 30% of striatal MSNs are spontaneously firing APs, which is in the accordance with previous findings (Dehorter et al., 2011). During development, MSNs are generated by two waves of neurogenesis, separated by 2 days in mice (Passante et al., 2008; Martin-Ibanez et al., 2017). This time gap persists during the postnatal development, and gives rise to two principal striatal compartments: patches and matrix (Brimblecombe and Cragg, 2017). The functional segregation of developing MSNs in P6–7, observed by us and others, may reflect the different time of neurogenesis and delayed development of spontaneously active cells. This is indirectly confirmed by relatively less developed RMP and evoked AP firing in “active” cells, comparing to “silent” cells and MSNs of adult DS.

In GlyRα2KO animals, both spontaneous and current-induced AP firings were severely affected in spontaneously active MSNs of 6–7 days-old mice. Genetic loss of functional GlyRs or pharmacological blockade of GlyRs led to broadening of individual AP and decrease in frequency of spontaneous APs in active MSNs. The observed effect cannot be attributed to changed function of NMDARs. AP firing by “silent” MSNs, which may be at more advanced stage of development, was not affected, as well as AP shape in the adult animals (Molchanova et al., 2018). These findings suggest that GlyRs activate some yet unknown mechanism, specific for neonatal neurons, and shift to more classical extrasynaptic transmission later in development.

This neonatal mechanism of regulation of the AP shape by GlyRs is still unclear. Decrease in the amplitude and broadening of the APs are usually associated with loss of voltage-dependent potassium currents, mediated by either Kv or BK/SK channels (Bean, 2007; Kimm et al., 2015). Both Kv and Ca-activated potassium channels are voltage-dependent, and increase the conductance upon membrane depolarization. In our experiments, potassium current evoked by depolarising voltage steps did not differ between spontaneously active neonatal WT and GlyRα2KO MSNs, which rule out the involvement of voltage-activated potassium channels in the GlyR-mediated regulation of AP shape. However, in neonatal GlyRα2KO MSNs we found the strong decrease in the potassium current, evoked by hyperpolarization. This current is mostly mediated by inward-rectifying potassium channels, for example Kir2 (classic inward rectifiers) and Kir3 (G-protein-gated potassium channels, GIRKs) (Hibino et al., 2010). Kir2 channels are abundantly expressed by adult MSNs, producing their hyperpolarized RMP, but the expression of these channels starts only at postnatal day 10 (Dehorter et al., 2011). GIRKS are absent in adult striatum, but are moderately expressed during late embryonic and early postnatal development. Interestingly enough, we did not find any difference in inward-rectifying potassium current in P14 MSNs, which match the developmental regulation of GIRK expression, and correlate with the temporary effect of GlyRs on AP firing. These facts make GIRKs a good candidates for downstream effectors of GlyR activation in developing MSNs. However, the physiological role of GIRKs is mostly linked to regulation of RMP and rheobase, and not to AP shape (Ehrengruber et al., 1997; Koyrakh et al., 2005). Together, our data suggest that α2 subunit GlyRs mediate regeneration of the AP firing in developing spontaneously active MSNs, with possible involvement of some inward-rectifying K^+^ channels. Such mechanism has not been shown before for GlyRs. Since we did not detect endogenous phasic or tonic glycinergic current in MSNs at this developmental stage, it might be possible that GlyRs act though some non-canonical mechanism, similar to other ligand-gated ionotropic receptors which may activate G protein-mediated pathways (Valbuena and Lerma, 2016). However, further studies are required to prove this concept.

Since GlyRs regulate the neonatal activity of MSNs, this might have long-term consequences on the formation and function of the striatal network in general. We confirmed that GlyRα2KO mice have impaired development of glutamatergic, but not GABAergic, synapses onto striatal MSNs. A key feature of glutamatergic synaptic maturation is an increase in the AMPAR-mediated component of the post-synaptic current. Only after adequate rise in intracellular Ca^2+^, AMPARs will be inserted into the synapse (Hell, 2016). This synaptic maturation, evaluated by the AMPA-mEPSCs, is reduced at early postnatal stages of the MSNs which implies that in the GlyRα2KO mice, a reduction of functional synapses is present (Bekkers and Stevens, 1995). We saw no effect of pharmacologically blocking GlyRs on these synaptic events indicating that no acute mechanism is involved in this observation. The behavioral data also seems to indicate a change in the striatal activity and connectivity already present at early postnatal stages. Spontaneous activity and glutamatergic innervation have both been found to cause changes in USV and time to right (Araujo et al., 2015; Chen et al., 2016). Here, we state that this connectivity defect is of a postsynaptic (dorsal striatum MSNs) origin rather than a presynaptic origin (cortical). Both at the functional (paired-pulse ratio) and molecular (VGluT1 protein expression) levels, we saw no change in cortical afferents. Considering the fact that we did not detect a reduction of the dendritic spines of the MSNs in all developmental stages, we evaluated the composition of the glutamatergic cortico-striatal synapses. This is further strengthened by findings in earlier work in which the connectivity defects were of intra-cortical nature. No evidence was found that pyramidal neurons projecting to extra-cortical structures were morphological or functionally affected in the GlyRα2KO animals (Morelli et al., 2016). The decreased ratio of AMPA/NMDA in GlyRα2KO mice suggests inadequate maturation at the post-synaptic level, implying a relatively higher number of silent synapses in GlyRα2KO animals. This deficiency in maturation probably starts very early in development, since the frequency of NMDAR-mediated mEPSCs was not affected in neonates. Decrease in the frequency of AMPAR-mediated mEPSCs, together with the same frequency of NMDAR-mediated mEPSCs in P6-7 MSNs indirectly shows a relatively higher number of silent synapses in KO animals.

Our findings on the development of connectivity defects in GlyRα2KO mice could in part explain the involvement of the GlyR α2 subunit mutations in the pathology of ASD and more specifically, the motor phenotypes present in these patients. ASD are characterized by two main symptom domains – deficits in social interactions and restrictive, repetitive patterns of behavioral output (Fuccillo, 2016; Sungur et al., 2017). Dysfunction of the dorsal striatum is postulated to underlie the motor dysfunctions seen in patients with ASD (Arriaga et al., 2012; Shepherd, 2013). The striatum is a hub processing the sensory information for the realization of voluntary movements. Considering the high level of excitatory convergence (roughly 10:1) into the striatum, initial processing of action selection occurs at these synaptic connections (Yim et al., 2011). Deficits in these synaptic glutamatergic inputs into the dorsal striatum have been linked to repetitive behaviors in ASD (Yin, 2010). Our findings are in line with other works on animal models of ASD, showing that ASD phenotypes are often attributed to changes in glutamatergic cortical projections and consequently their synaptic integration into downstream brain regions such as the dorsal striatum (DS) (Peca et al., 2011; Tsai et al., 2012; Peixoto et al., 2016; Sungur et al., 2017).

Taken together, our findings suggest that GlyRs containing α2 subunit promote the early immature spontaneous activity of MSNs by facilitating the regeneration of membrane potential after AP firing. We also demonstrate that proper spontaneous activity is needed for correct maturation and function of the glutamatergic inputs into the dorsal striatal MSNs. When GlyRα2 function is lost, glutamatergic synapse function stays disturbed in adulthood, which is probably caused by impaired spontaneous activity of MSNs during early development. This highlights the importance of proper network formation in the dorsal striatum, which can be influenced from an early developmental stage onward.

## Author Contributions

SS, J-MR, BB, DG, JC, and SM designed the experiments. JC and SM performed the experiments. RH generated the Glra2-KO. JC, JD, and SM analyzed the data. JC and SM wrote the article. VB, SB, and GM performed the ultrasonic vocalizations experiment and CB assisted with the analysis. All authors revised the article and agreed to be accountable for all aspects of the work.

## Conflict of Interest Statement

The authors declare that the research was conducted in the absence of any commercial or financial relationships that could be construed as a potential conflict of interest.
