# Supplementary material for: Alpha2-Containing Glycine Receptors Promote Neonatal Spontaneous Activity of Striatal Medium Spiny Neurons and Support Maturation of Glutamatergic Inputs
Source: Front Mol Neurosci. 2018 Oct 15;11:380. doi: 10.3389/fnmol.2018.00380 (PMC6196267; doi:10.3389/fnmol.2018.00380)
Supplement: Supplementary file 1 [file Data_Sheet_1.pdf]

Supplementary Table 1. Passive membrane properties and intrinsic excitability parameters of silent neonatal MSNs from wild-type and GlyRα2KO mice.

	Silent MSNs, WT P5-8	Silent MSNs, GlyRα2KO P5-8	P value
**Input resistance (MΩ)**	1426±174.0	1797±239.8	0.085
**Capacitance (Cm)**	69.59±5.17	73.81±13.43	0.831
**Resting membrane potential, mV**	-69.55±2.00	-74.79±3.21	0.159
**Rheobase (pA)**	27.92±3.30	21.82±1.82	0.354
**Threshold (Vm)**	-36.32±2.34	-37.16±2.61	0.992
**Holding current to -70 mV (pA)**	-2.96±1.91	2.27±2.35	0.203
**Slope of evoked firing rate (Hz/pA)**	0.59±0.05	0.72±0.04	0.105
**Accommodation current (pA)**	135.2±13.27	121.0±20.19	0.538
**AP amplitude (mV)**	59.65±2.48	54.05±3.7	0.25
**AP half-width (ms)**	2.57±0.24	2.40±0.31	0.53
**Fast AHP, half-width (ms)**	24.87±1.05	22.73±1.84	0.26
**Fast AHP, amplitude (mV)**	-6.45±0.33	-7.05±0.63	0.28
**N**	24	11	

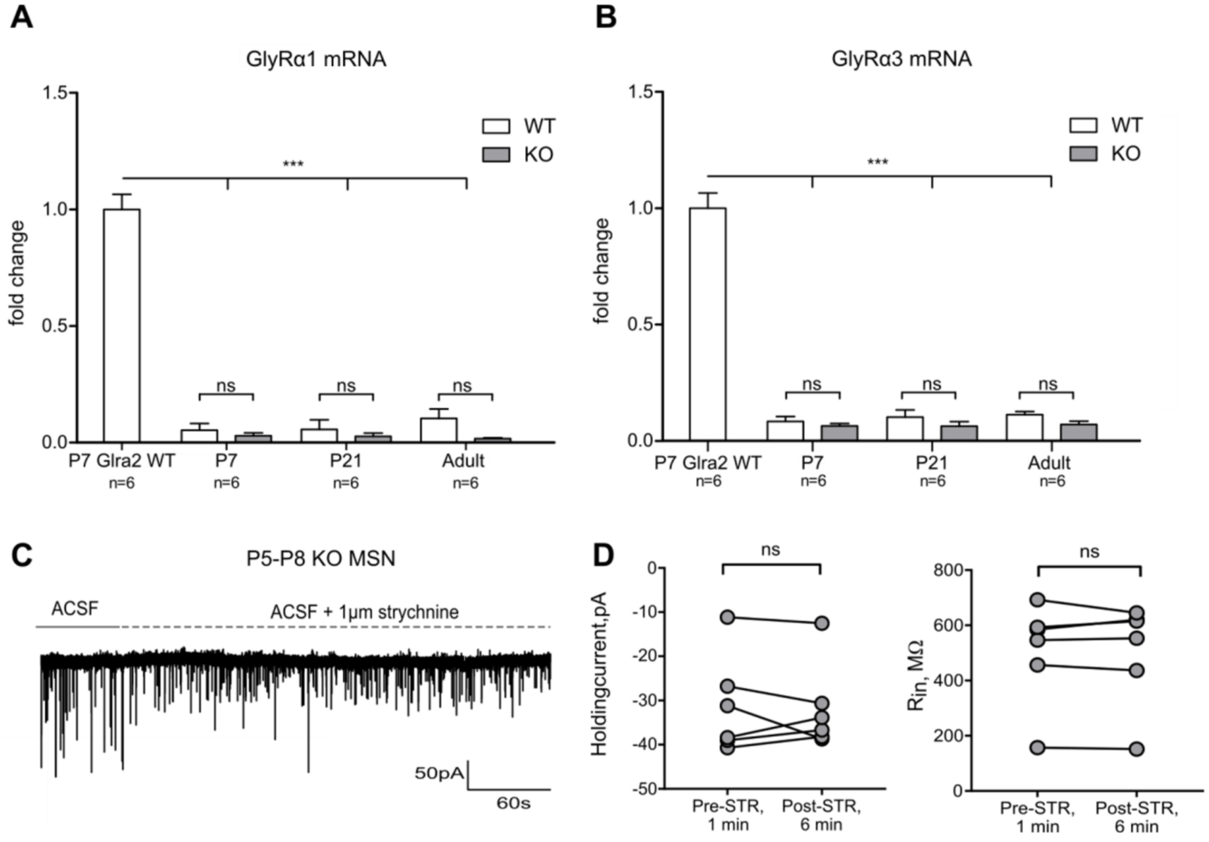

Supplementary Figure 1. GlyR α1 and GlyR α3 subunit mRNAs are barely detectable in dorsal striatum of wild-type and knockout mice. A,B) Q-PCR results, plotted as ΔΔCt, normalized to the expression level of the GlyR α2 subunit mRNA in dorsal striatum of P7 wild-type animals. C) Representative trace of measuring tonic glycinergic currents in GlyR α2 KO MSNs. Baseline was measured after 1 min of aCSF perfusion followed by 5 min of aCSF + 1µM strychnine perfusion. D) Holding current (Pre-STR: -40.74±5.497pA; Post-STR:-41.68±6.034 pA) and input resistance (Pre-STR: 505±76.2 MΩ; Post-STR: 503.7±76.75 MΩ) was plotted before (Pre-STR) and after (Post-STR) strychnine application.

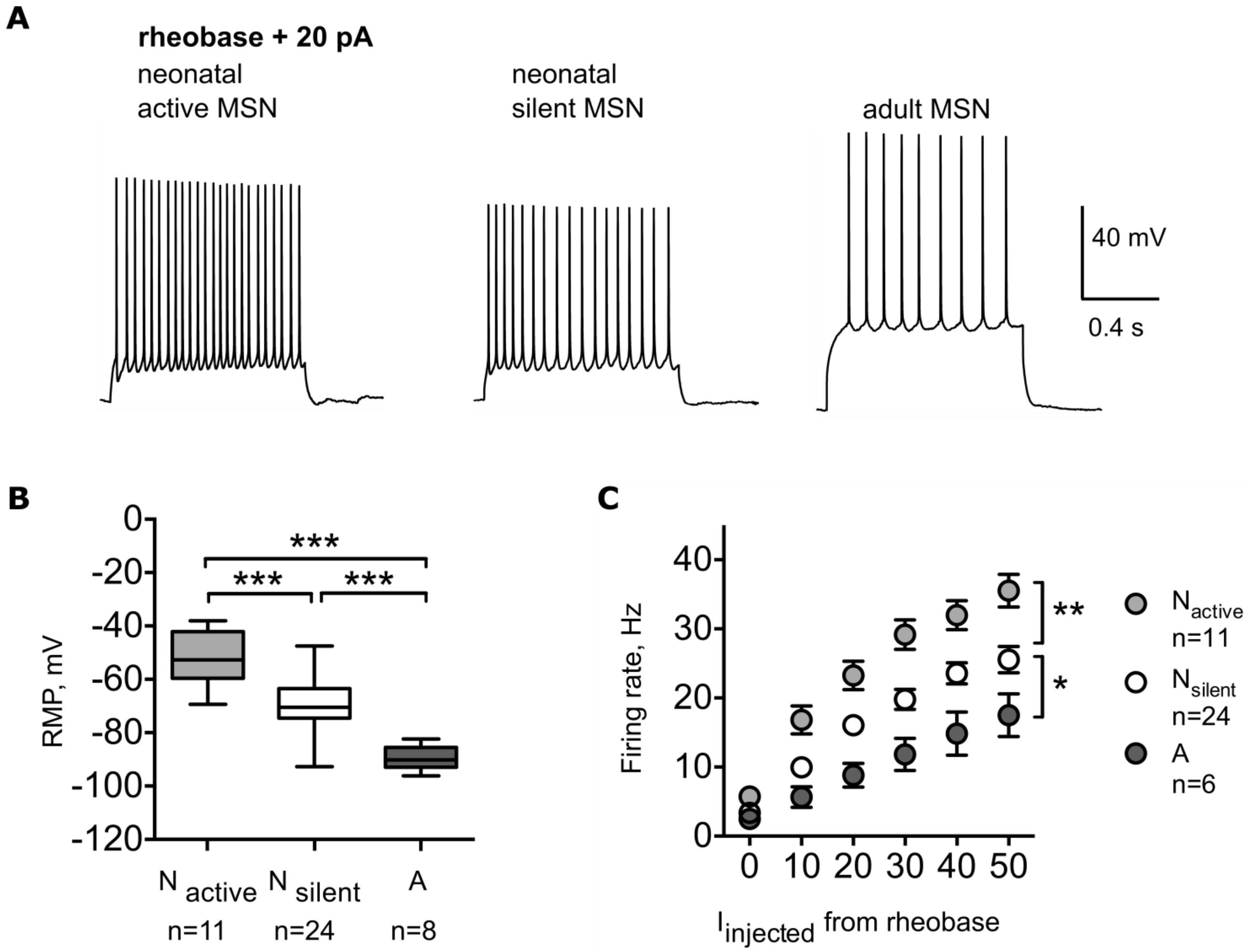

Supplementary Figure 2. Evoked action potentials in neonatal active, silent and adult MSNs. A) Example traces of evoked action potentials recorded from WT mice of either neonatal active, silent and adult MSNs. B) Graph depicting the resting membrane potential (RMP) of the different MSN populations investigated. C) Firing frequency plot from WT mice of either neonatal active, silent and adult MSNs. Data are presented as box plot (whiskers indicate variability from minimum to maximum) and mean±SEM, *p<0.05, ** p<0.005, ***p<0.001.

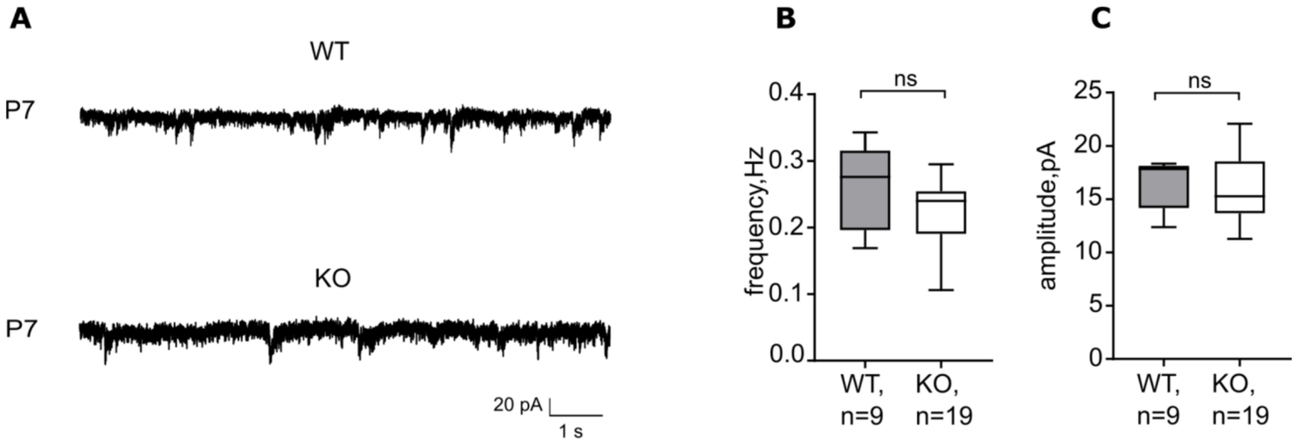

Supplementary Figure 3. **NMDA-mEPSCs are not affected by the loss of the GlyR α2 subunit**. A) Continuous recording including traces of NMDA-mEPSCs. B,C) Both frequency (WT: 0.258±0.021 Hz; GlyRα2KO: 0221±0.013 Hz) and amplitude (WT: 16.45±0.752 pA; GlyRα2KO: 15.97±0.699 pA) did not significantly differ ($p<0.05$). Data are presented as box plot (whiskers indicate variability from minimum to maximum).

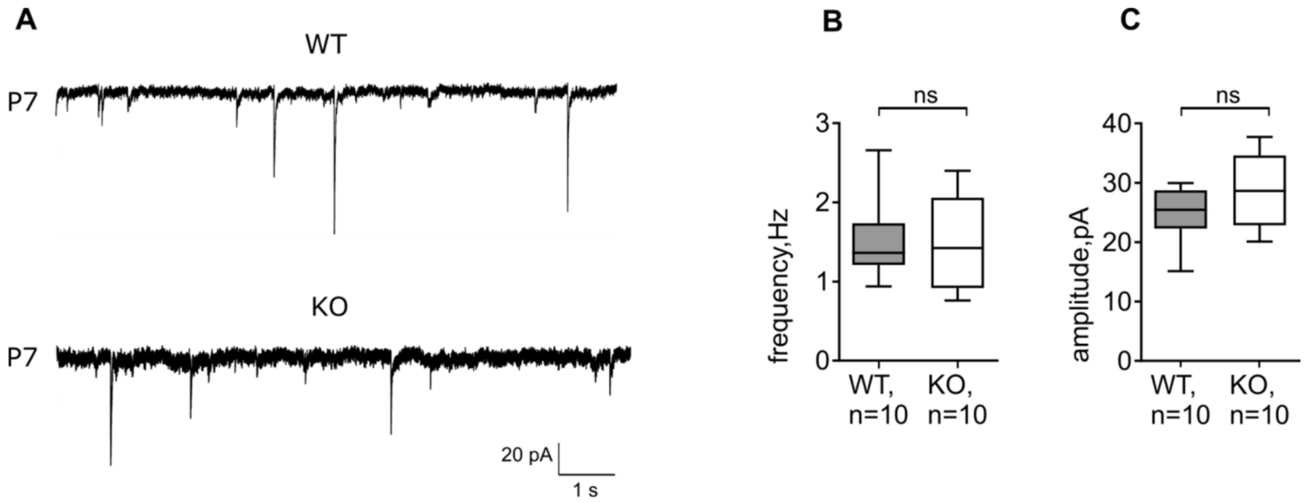

Supplementary Figure 4. GABAergic innervation of MSNs is not affected in GlyRα2KO mice. A) representative traces of mIPSCs in P5-P8 WT and GlyRα2KO MSNs. B,C) Both frequency (WT: 1.52±0.2 Hz; GlyRα2KO: 1.48±0.16 Hz) and amplitude (WT: 34.60±2.23 pA; GlyRα2KO: 30.72±1.82 pA) did not significantly differ ($p<0.05$). Data are presented as box plot (whiskers indicate variability from minimum to maximum).

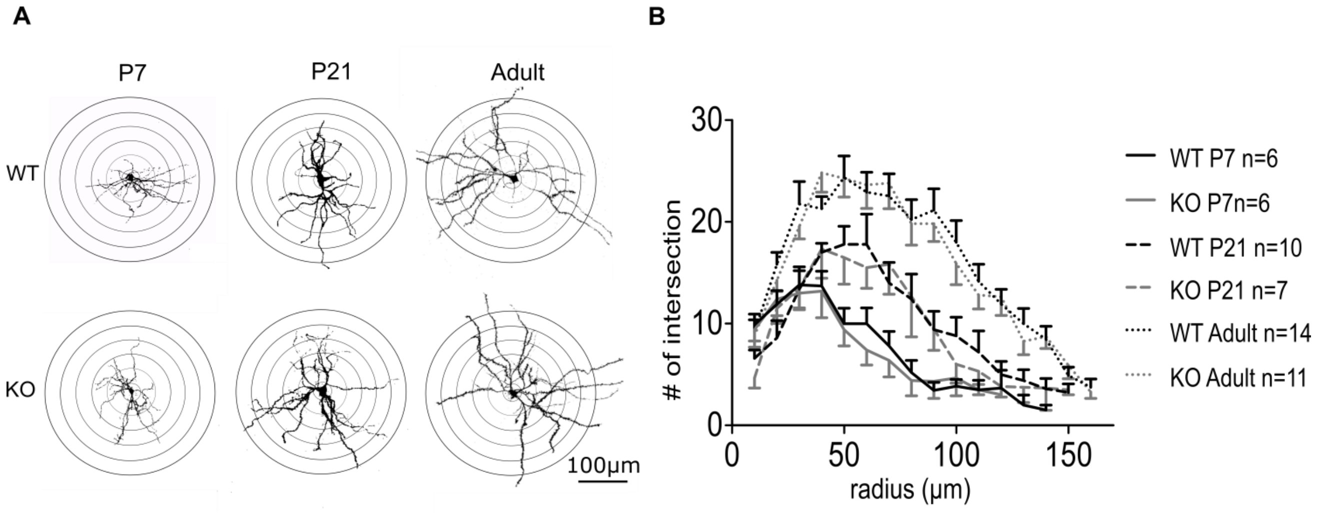

Supplementary Figure 5. Deletion of the GlyR α2 subunit does not affect the morphology of dendritic tree in MSNs of different ages. A. Example images of dendritic trees of MSNs in WT and KO animals of different ages. B. Quantification of Sholl analysis of MSN dendritic trees. Data are presented as mean ± SEM.
